# Items of General Interest

**Published:** 1915-04

**Authors:** 


### Items of General Interest.

It is good news and many physicians will be glad to know that the moral and steadfast principles of the Martin H. Smith Company in maintaining Glyco-Heroin (Smith) for the exclusive use of physicians is very commendable. The composition is not being changed to meet any of the exemptions or privileges allowed under the "Harrison Anti-Narcotic Law," whereby it might be sold to the public. It has always been, and shall continue to be, a stable, uniform and dependable product for the convenience and use of physicians only, in the treatment of cough, bronchitis, whooping cough, etc. It is not necessary for the physician to use special prescription blanks in prescribing, nor is a copy or record required, a fact well to be remembered in its favor.

### DR. DOWLING WEDS IN MONROE, LA.

The TEXAS MEDICAL JOURNAL is pleased to learn of the marriage of Dr. Oscar Dowling, famous president Louisiana Board of Health and State Health Officer, and a member of the editorial staff of the "Red Back." The public has been regarding Dr. Dowling as a confirmed bachelor. The prolonged bachelor days were ended at 5 o'clock on the afternoon of March 16, when he was married to Mrs. Lulu Tendal George at the home of her parents in North Second Street, Monroe, La., by Rev. C. S. Newman, pastor of the First Presbyterian Church. The JOURNAL'S best wishes are extended Dr. and Mrs. Dowling.

An institution of which the people of Cameron and Milam county are justly proud and which deserves the patronage of those in need of medical attention is the new Cameron Sanitarium. It is modern, well located and has a corps of well trained physicians and surgeons, of whom Dr. Rischar is chief.

A charge of violating the Harrison Narcotic Law was filed in Dallas, March 12, by Deputy Internal Revenue Collector G. D. Hunt against Wallace Chiek and P. E. Dugan, who was a fourth-year medical student at the Baylor Medical School in Dallas. Wallace Chiek, who was joined with Dugan in the complaint, was given a hearing and his bond was fixed at $1500.

Dr. S. M. D. Clark addressed the Fifty-sixth District Medical Society in San Antonio on March 16, on the subject of "Cancer and Its Treatment," telling of a new method which has been successfully employed in cases hitherto by many considered incurable. The address caused quite a sensation.

Probably 2000 physicians from all over Texas will attend the annual convention of the State Medical Association in Fort Worth May 4, 5 and 6. It is promised by the committees in charge that all will derive great benefit from one of the best programs ever arranged for the association's annual meetings. There will be meetings at the First Baptist Church, Temple Beth-Eli and the Chamber of Commerce. It is probable that the House of Delegates will hold its meetings in the Chamber of Commerce basement auditorium. The main place of meeting will be, however, at the First Baptist Church. This building and the Temple Beth-Eli, where scientific section work will be conducted, were chosen because of their proximity to the Chamber of Commerce.

Dr. W. C. Rucker is among the many prominent national men who have made known their intention of attending the State meeting in Fort Worth. The doctor is assistant surgeon general of the United States Public Health Service, and is the man who stopped the plague in New Orleans last summer. The doctor will come from Washington to attend the meeting.

The National Tubercular Sanitarium Association of San Antonio is requesting help and assistance in building and maintaining a hospital for the charitable treatment of persons afflicted with tuberculosis. J. Feeney of San Antonio, representing the association, which is headed by a board of directors composed of men of highest standing, proposes to found the sanitarium by popular subscription. The proposed site is about thirty miles from the city of San Antonio.

Dr. Henry B. Deckard, a noted specialist on children's diseases, delivered a splendid talk in Dallas recently before the Associated Mothers' Clubs. His topic was, "Why is the Child Nervous?" He greatly impressed his audience.

The effect of the enforcement of the Harrison Anti-Drug Act is now being watched closely by the police department of every city in the United States. The new law became effective March 1, and each day since that time it is becoming more difficult for those addicted to the use of morphine, cocaine, heroin or opium to secure the drugs.

After the ordinance creating the position of visiting nurse for the city and an advisory board to act with the city government in directing the work had been passed under suspended rules at the meeting of the city commission of Waco, March 22, Mayor Riggins announced that he would veto the ordinance within the ten days allowed by the charter provision.

Dr. Isaac A. Withers of Fort Worth told the Ad Men's Club in Dallas at a luncheon on March 10 why doctors do not advertise. He said in part: "The only thing that could be properly advertised would be that we could cure them as soon as possible, but make no guarantee against their resistance or the extent of their illness."

The JOURNAL is in receipt of a handsome program of the thirty-seventh semi-annual meeting of the South Texas Medical Association, to be held in Victoria on April 8 and 9. We regret that limited space prevents our printing this program in full.

### Change of Location.

Dr. J. W. Brown, formerly of Bremond, is now located at Pearsall.

Dr. E. M. Wood, who has been living in Georgetown, is now practicing in Hutto.

Dr. W. G. Carter has moved from Ellis county to the city of Quanah.

Dr. J. H. Hendrix has located in Bynum, going there from Edgewood.

Dr. J. E. Williamson, who has been living in Chandler, has located at Noonday.

Dr. E. K. Crockett, recently of San Antonio, has located in Uvalde.

